# Label-free separation of peripheral blood mononuclear cells from whole blood by gradient acoustic focusing

**DOI:** 10.1038/s41598-024-59156-7

**Published:** 2024-04-16

**Authors:** Julia Alsved, Mahdi Rezayati Charan, Pelle Ohlsson, Anke Urbansky, Per Augustsson

**Affiliations:** 1 AcouSort AB, Medicon Village, S-223 81 Lund, Sweden; 2 Department of Biomedical Engineering, Lund University, Ole Römers väg 3, 22363 Lund, Sweden

**Keywords:** Biomedical engineering, Fluid dynamics, Acoustics, Microfluidics

## Abstract

Efficient techniques for separating target cells from undiluted blood are necessary for various diagnostic and research applications. This paper presents acoustic focusing in dense media containing iodixanol to purify peripheral blood mononuclear cells (PBMCs) from whole blood in a label-free and flow-through format. If the blood is laminated or mixed with iodixanol solutions while passing through the resonant microchannel, all the components (fluids and cells) rearrange according to their acoustic impedances. Red blood cells (RBCs) have higher effective acoustic impedance than PBMCs. Therefore, they relocate to the pressure node despite the dense medium, while PBMCs stay near the channel walls due to their negative contrast factor relative to their surrounding medium. By modifying the medium and thus tuning the contrast factor of the cells, we enriched PBMCs relative to RBCs by a factor of 3600 to 11,000 and with a separation efficiency of 85%. That level of RBC depletion is higher than most other microfluidic methods and similar to that of density gradient centrifugation. The current acoustophoretic chip runs up to 20 µl/min undiluted whole blood and can be integrated with downstream analysis.

## Introduction

Peripheral blood mononuclear cells (PBMCs), i.e. lymphocytes and monocytes, as a subgroup of the white blood cell (WBC) population, are critical components of the immune system. They are responsible for activating and regulating immune responses to pathogens and are frequently targeted for numerous diagnostic, therapeutic, and research purposes. Separating these cells from whole blood is often a first step towards performing complex analyses and treatments such as measuring cytokine release^1–3^, genome sequencing^4–6^, and cell therapies^7,8^. Due to their large and versatile area of usage, separating PBMCs from patient blood is common in many laboratories. Density gradient centrifugation is perhaps the most common traditional method to isolate subpopulations of WBCs from blood. Cells sediment through layers of liquids of different densities formed by gradient media, such as Ficoll-Paque^9,10^ or Histopaque^11,12^, until reaching their points of neutral buoyancy. Drawbacks with this method are that (*i*) the accuracy is limited by the difficulties of accurately collecting the thin sheaths of layered cells from the test tube for cells of contiguous densities, (*ii*) it cannot easily be implemented for applications of handling small sample volumes, and (*iii*) it is challenging to implement in a flow-through format for in-line integration in compact automated analytical instrumentation to facilitate diagnosis or treatment of a patient at the point-of-care.

Over the past decade, microfluidic techniques for cell separation have been established as an alternative to macro-scale approaches. Microfluidics offers advantages such as minimizing manual procedures, reducing processing time, handling smaller sample volumes, higher accuracy, and, notably, label-free separation of target cells based on their biophysical properties^13–15^. Different biophysical properties such as size^16–19^, density^20,21^, deformability^22,23^, electrical impedance^24,25^, and magnetic^26,27^ and optical properties^28^ in different forms have been investigated to improve the separation performance in terms of efficiency, purity, and throughput. However, due to the extremely high number of cells in the blood (typically 5 × 10^9^ cells/µl) and its non-Newtonian fluid properties, it is with most methods challenging to process undiluted blood in a microchannel. Additionally, target PBMCs have a low abundance compared to the vast background of red blood cells (RBC) with a ratio of approximately 1:1000, which makes them difficult to isolate. Among various microfluidic methods, inertial^29,30^ and viscoelastic^31^ separation, microfiltration^32^, and deterministic lateral displacement^33^ have been reported with enhanced performance parameters; however, they still face some challenges. Inertial microdevices utilize selective lysis of RBCs or use a highly diluted blood sample before fractionation of WBC subpopulations since high cell concentrations are associated with poor separation due to hydrodynamic interactions between cells. Viscoelastic focusing can cause contamination due to the dilution of target samples with a viscoelastic fluid, which may hinder further processing. Microfiltration and deterministic lateral displacement devices are prone to clogging^34^. To summarize, most current techniques need to remain at very low throughput, require many-fold diluted blood, or do not achieve high purity of the produced PBMCs fraction.

The present study deals with acoustophoresis as a separation technique to address the current challenges in directly separating PBMCs from undiluted blood. Acoustophoresis is a gentle and non-invasive tool where suspended cells are separated in an ultrasonic field and has been extensively demonstrated for a wide range of application areas such as stem cells^35^, circulating tumor cells (CTCs)^36–41^, WBCs^42,43^, generating plasma from whole blood^44^, removal of platelets from peripheral blood progenitor cells^45^, and separating bacteria from blood components^46,47^. A piezoelectric transducer generates vibrations in a glass or silicon chip and a standing wave forms across a microfluidic channel with a pressure node in the center plane and anti-nodes at the channel walls. The sound field scatters on the cells, leading to a force and resulting in migration of the cells towards the node or anti-node. The cell velocity is determined by its size, density, and compressibility in relation to the surrounding liquid’s density and compressibility. Cell populations of different sizes, densities, and compressibilities can thus be separated from each other in a label-free fashion. To improve the separation performance, the density and compressibility of the suspending medium can be altered, thereby tuning the acoustic contrast of the cells such that the migration of one cell type is reduced or changes direction^48,49^. To separate PBMCs from RBCs and granulocytes, Urbansky et al.^42^ optimized the suspending medium by pre-mixing different concentrations of Percoll (a standard component to alter the density of cell media) into the input sample and thereby changing the acoustic mobility of cells. PBMCs were successfully enriched relative to RBCs by a factor of 2,800 with a PBMC recovery of up to 88%. Similar to other studies, a drawback of this work was a 20 × dilution of the blood. Performing cell separation by having cells migrating into a medium of gradually increasing acoustic impedance (*Z*_*m*_), can significantly improve the accuracy due to stabilizing acoustic body forces acting on the medium^41,50–53^, and higher concentrations of cells can be processed at maintained performance due to less hydrodynamic interactions between cells in the stabilized medium^54,55^.

**Figure 1:**
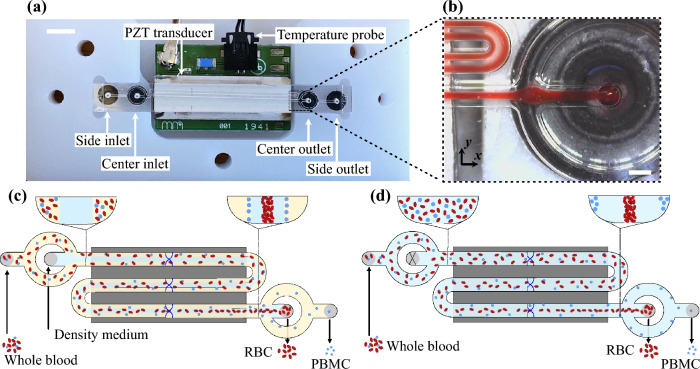
Overview of the acoustic separation of PBMCs from whole blood. (**a**) Photograph of the chip placed in its holder. (**b**) Camera view of RBCs packed in the center of the channel. The principles for isolating PBMCs from whole blood by (**c**) processing unmodified blood and introducing a barrier medium in the separation channel and (**d**) modifying the density and compressibility of the plasma before the separation. The scale bars are in (**a**) 5 mm, and in (**b**) 200 μm.

Herein, we present two approaches for flow-through separation of PBMCs from undiluted or minimally diluted blood utilizing a commercial instrument for acoustophoresis-based cell separation, Fig. 1. In the first approach, which is novel, undiluted blood is processed, and a barrier medium is introduced through a separate inlet to the chip, allowing only RBCs and granulocytes to migrate into the barrier medium, whereas PBMCs remain at the interface between the blood plasma and the barrier medium. In the second approach, similar to Urbansky et al.^42^ but for much lower dilution factors, the blood is pre-mixed with a medium that alters the acoustic properties of the blood plasma before the separation such that PBMCs gain negative contrast and thus move away from RBCs and granulocytes.

## Materials and methods

### Cell preparation and blood samples

Blood with hematocrit levels between 39% and 46%, measured using a hematocrit centrifuge (Haematokrit 210, Andreas Hettich GmbH & Co. KG, Germany) was obtained from healthy volunteers at Lund University Hospital (Lund, Sweden) and collected in vacutainer tubes with ethylenediaminetetraacetic acid (EDTA) as an anticoagulant (BD Bioscience, San Jose, CA, USA). The blood was drawn with written informed consent, following the Helsinki Declaration, and was approved by the Swedish ethical review authority (ref. no. 2020–05,818). PBMCs and neutrophils were isolated directly from blood samples using EasySep Direct Human PBMC and Neutrophils Isolation Kit (STEMCELL Technologies, Norway), respectively. Collected PBMCs and neutrophils were washed and resuspended in phosphate-buffered saline (PBS, Sigma-Aldrich) containing fluorescent dye. Two types of fluorescence dyes were used to stain cells for microscopy imaging: CellTracker Green CMFDA (Thermo Fisher Scientific) and CellTracker Red CMTPX (Thermo Fisher Scientific). Cells were incubated at 37 °C for 20 min in PBS with 1 μM of fluorescence dye. Finally, after two centrifugation steps, the cells were resuspended in PBS with 2% fetal bovine serum (FBS) at a concentration of 1 × 10^6^ cells per ml and stored on ice before the experiments.

### Isotonic optiprep solutions

OptiPrep (STEMCELL Technologies, Norway) containing high concentrations (60% w/v) of the dense molecule iodixanol was chosen as a medium to modify the density (*ρ*) and compressibility (*κ*) of the suspending media due to its low ratio of viscosity to acoustic impedance^50^ *Z*_*m*_ = √(*ρ/κ*). Density-adjusted media were prepared by adding a required amount of the OptiPrep to PBS to form an isotonic solution. The isotonic stock solution was prepared in two steps: first, 10 × PBS was diluted 6 times to reach an osmolarity of 500 mOsm/L, and second, 65%_vol_ OptiPrep was mixed with 35%_vol_ of the diluted PBS, resulting in a stock solution containing 39% iodixanol of osmolarity around 300 mOsm/L. Working solutions were prepared by diluting the stock solution in the standard 1 × PBS, as listed in Table 1. The density and compressibility of each working solution were measured by a combined density and sound velocity meter (DSA 5000 M, Anton Paar GmbH, Graz, Austria) and used to calculate the acoustic impedance through the relation *Z*_*m*_ = *ρc*, where *c* is the sound velocity.

**Table 1:** Isotonic working solutions used in the experiments.

Iodixanol concentration	Barrier acoustic impedance	Pre-mix acoustic impedance	Vol. PBS	Vol. working solution
24%	1.690 MPa s/m	1.657 MPa s/m*	625 µl	1 ml
27%	1.713 MPa s/m	1.672 MPa s/m	444 µl	1 ml
30%	1.736 MPa s/m	1.687 MPa s/m*	300 µl	1 ml
33%	1.759 MPa s/m	1.703 MPa s/m	182 µl	1 ml
36%	1.782 MPa s/m	1.719 MPa s/m*	83 µl	1 ml
39%	1.806 MPa s/m	1.735 MPa s/m	0 µl	1 ml

*calculated from linear fitting. Pre-mix acoustic impedances were measured for mixtures of whole blood (40%_vol_) and the working solution (60%_vol_), whereafter RBCs were removed.

### Cell separation platform

The separation presented in the current study was performed in a commercial instrument for acoustic cell separation (AcouWash, AcouSort AB, Sweden), a benchtop research system for sample purification and cell separation through continuous flow acoustophoresis. The acoustic chip [Fig. 1a] in the instrument contains a separation channel which is optimized for 2 MHz resonance and has a trifurcation at each end, where the two side streams and central flow join and split. A piezo-ceramic transducer is located underneath the chip. The chip was actuated at maximum power, and the temperature was regulated to avoid drift in the resonance frequency. Flow regulation was carried out by regulating the air pressure in the liquid containers. The cell separation platform has an internal volume of ~ 50 µl for the flow path of the PBMCs which leads to an expected loss of ~ 10% of the sample if the processed sample volume is 500 µl.

### Flow settings

The separation of PBMCs from blood was carried out for two different configurations: the *barrier medium* and the *pre-mix* approach. In the barrier medium approach, Fig. 1c**,** the blood sample is introduced in the side inlet of the separation channel while the barrier medium is injected in the center. In the pre-mix approach, Fig. 1d, the central inlet is set to 0 µl/min (blocked), and the sample flows into the channel from the side inlet. At the end of the separation channel, the flow is split into three branches, and the two side branches are thereafter recombined in a common side outlet. Table 2 summarizes the flow rate (Q) settings for the two separation configurations. The average retention time of a cell in the separation channel was ~ 1 s. When imaging cells by microscopy, the sample flow in the side inlet was instead regulated using a pressurized tube holder set to 2 bar (Elveflow adaptor for Falcon© tubes). Two syringe pump units (Tricontinent C-series, Gardner Denver, USA) were programmed to control the flow to the central inlet and to collect the entire flow from the outlets.

**Table 2:** Summary of the two configurations for acoustic separation of PBMCs.

Configuration	Side inlet	Central inlet	Central outlet	Side outlet
Barrier medium	0.5 ml of undiluted whole blood. **Q = 20 µl/min**	Solutions in Table 1. **Q = 40 µl/min**	Focused RBCs + granulocytes. **Q = 40 µl/min**	Plasma + PBMCs. **Q = 20 µl/min**
Pre-mix	40% undiluted whole blood + 60% solutions in Table 1. Q = **50 µl/min**	**Q = 0 µl/min** (blocked)	Focused RBCs + granulocytes. **Q = 30 µl/min**	Plasma + PBMCs. **Q = 20 µl/min**

Q is the flow rate.

### Microscopy

To visualize the acoustophoretic motion of cells, the chip was mounted upside down in an inverted microscope (Eclipse Ti2, Nikon, Tokyo, Japan) equipped with a CMOS camera (Prime 95B, Teledyne Photometrics, Tucson, Arizona). Stained PBMCs and neutrophils were spiked into the whole blood and imaged at the end of the separation channel. To monitor the density-adjusted medium at the channel outlet, we added a fluorescent tracer molecule (Dextran, Cascade Blue, 3000 MW, Thermo Fisher Scientific) under the assumption that iodixanol and dextran molecules diffuse at the same rate (D_iodixanol_ ≈ 2.5 × 10^–10^ m^2^ s^−1^ and D_dextran_ ≈ 2.2 × 10^–10^ m^2^ s^−1^). A laser illumination unit (Celesta light engine, Lumencor, OR, USA) was used with a multiband filter set (CELESTA-DA/FI/TR/Cy5/Cy7-A-000, Semrock optical filters, IDEX Health & Science) in three excitation channels with peak wavelengths at 365 nm, 488 nm, and 561 nm. The excitation channels and the camera were activated through external triggering.

When operating the chip outside of the AcouWash system the PZT transducer was driven by a function generator (AFG3022B, Tektronix, Inc., Beaverton, Oregon, USA) to deliver a resonant frequency (1.99 MHz) and different applied voltages as measured over the piezo with an oscilloscope (TDS1002, Tektronix, Inc., Beaverton, Oregon, USA).

### Immunostaining and flow cytometry enumeration

Output sample tubes were weighed before and after being processed in the AcouWash to determine the processed volume. To count WBCs, platelets, and granulocytes, both side and center output fractions were stained with the following conjugated monoclonal antibodies: CD45-PerCP (clone 2D1), CD61-PE (clone VI-PL2) and CD66b FITC (clone G10F5). To assess the purity of PBMCs, RBCs in the side outlet were stained with CD235a APC (clone GA-R2), and propidium iodide (PI) was used to indicate dead cells. All the material for flow cytometry analysis was purchased from BD Bioscience, San Jose, CA, USA. Stained samples were analyzed by BD FACS Canto II flow cytometer (BD Bioscience), and acquired data was further analyzed using the FCS Express software (De Novo Software**,** Pasadena, CA, USA).

### Definitions of separation-evaluation parameters

The intended function of the system is to take in undiluted or density-modified blood, guide PBMCs to the side outlet that collects the flow near both side walls, and guide RBCs and granulocytes through the central outlet. Cell separation efficiency for each cell type is defined as the number of cells of each kind in the side outlet fraction divided by the number of cells of that type in both outlet fractions combined. The side outlet PBMC purity is reported by two definitions: (i) the number of PBMCs divided by the number of all cells excluding platelets, and (ii) the number of PBMCs divided by the number of PBMCs and granulocytes (all WBCs). PBMC recovery is defined as the number of PBMCs in either the side, or both, outlets divided by the number of PBMCs in a sample of the same volume as the input sample, from the same test tube of blood.

## Theory

Size-independent separation of cells can be achieved by tailoring the properties of the suspending medium such that the magnitude and direction of cells’ motion are controlled. The time-averaged acoustic radiation force ^56,57^ acting on a suspended cell subjected to a one-dimensional standing pressure wave $p(y, t)={p}_{{\text{a}}}{\text{cos}}\left(ky\right)$ sin(2*πft*), with walls at *y* = 0 and *y* = *λ*/2, with the wave vector *k* = 2*π/λ*, wavelength *λ* and frequency *f*, can be described as

1 $${F}_{{\text{rad}}}=4\uppi \Phi k{E}_{{\text{ac}}}{\text{sin}}\left(2ky\right)$$

where the acoustic contrast *Φ* is given by

2 $$\Phi =\frac{1-\widetilde{\kappa }}{3}+\frac{\widetilde{\rho }-1}{2\widetilde{\rho }+1}$$

with the resulting velocity (*u*_rad_) of a cell

3 $${u}_{{\text{rad}}}=\frac{2{a}^{2}\Phi k{E}_{{\text{ac}}}}{3\eta }{\text{sin}}\left(2ky\right)$$

Here *E*_ac_ is the acoustic energy density, $\widetilde{\upkappa }={\upkappa }_{c}/{\upkappa }_{m}$ and $\widetilde{\rho }={\rho }_{c}/{\rho }_{m}$ are the relative compressibility and density of the cell with respect to the suspending medium. Thus, denser or less compressible cells than the medium will have positive acoustic contrast and move towards the pressure node, whereas cells of lower density or higher compressibility move to the anti-nodes due to a negative acoustic contrast.

A cell that moves through a medium of gradually changing density and compressibility will eventually reach a point where *Φ* = 0 and at this point *Z*_*c*_ ≈ *Z*_*m*_^50^. In short, for a cell to penetrate a medium in the direction towards the pressure node, its effective acoustic impedance *Z*_*c*_ = √(*ρ*_*c*_*/κ*_*c*_) must be higher than the local acoustic impedance of the medium.

## Results

**Figure 2:**
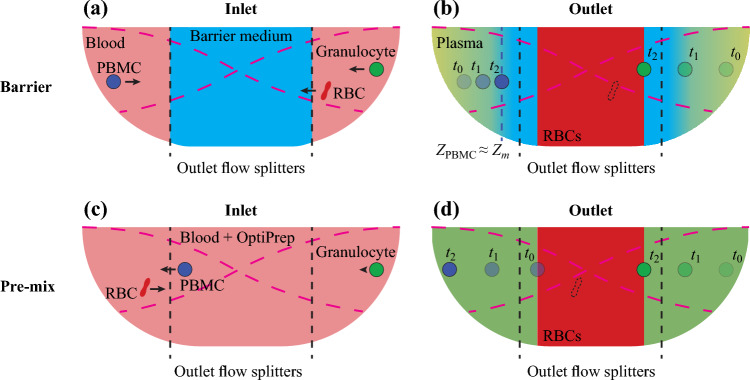
Illustrations of the separation schemes showing the (**a**) inlet and (**b**) outlet constellations for the barrier medium and the (**c**) inlet and (**d**) outlet constellations of the pre-mix approach. Dashed magenta curves represent the standing sound wave. Dashed black lines mark the locations of the flow splitters at the outlet between the central and side branches of the trifurcation.

In this work, we propose and evaluate two methods to isolate PBMCs from the whole blood based on a commercial instrument for acoustic cell separation. The mechanisms referred to as the *barrier medium* and the *pre-mix* approach are shown schematically in Fig. 2.

In the barrier medium approach, Fig. 2a and b, a cell-free solution containing iodixanol is injected through the central inlet of the chip to form an acoustic impedance barrier in the center of the microchannel. The barrier medium has higher *Z*_*m*_ than the initial cell medium and can only be penetrated by cells of sufficiently high acoustic impedance *Z*_*c*_, such as RBCs. As the RBCs pack up in the channel center, they displace the barrier medium such that it ends up on both sides of the RBCs. Since the solute molecules of the barrier medium are subjected to diffusion into the plasma region, the *Z*_*m*_ of the barrier gradually increases when approaching the RBC interface. Cells of low *Z*_*c*_, such as PBMCs or platelets, are prevented from crossing the barrier since their acoustic contrast becomes zero in the interface region between the plasma and the barrier medium. Subtypes of cells will end up in different locations inside the barrier depending on their acoustic impedances and can thus be recovered through separate outlets.

In the pre-mix approach, Fig. 2c and d, the acoustic contrast of the PBMCs is altered prior to processing them in the chip by supplementing the whole blood with a high-acoustic-impedance medium. Iodixanol was used to modulate the acoustic impedance with the intention of making PBMCs have negative acoustic contrast while RBCs and granulocytes should have positive contrast.

### Barrier medium PBMC enrichment

**Figure 3:**
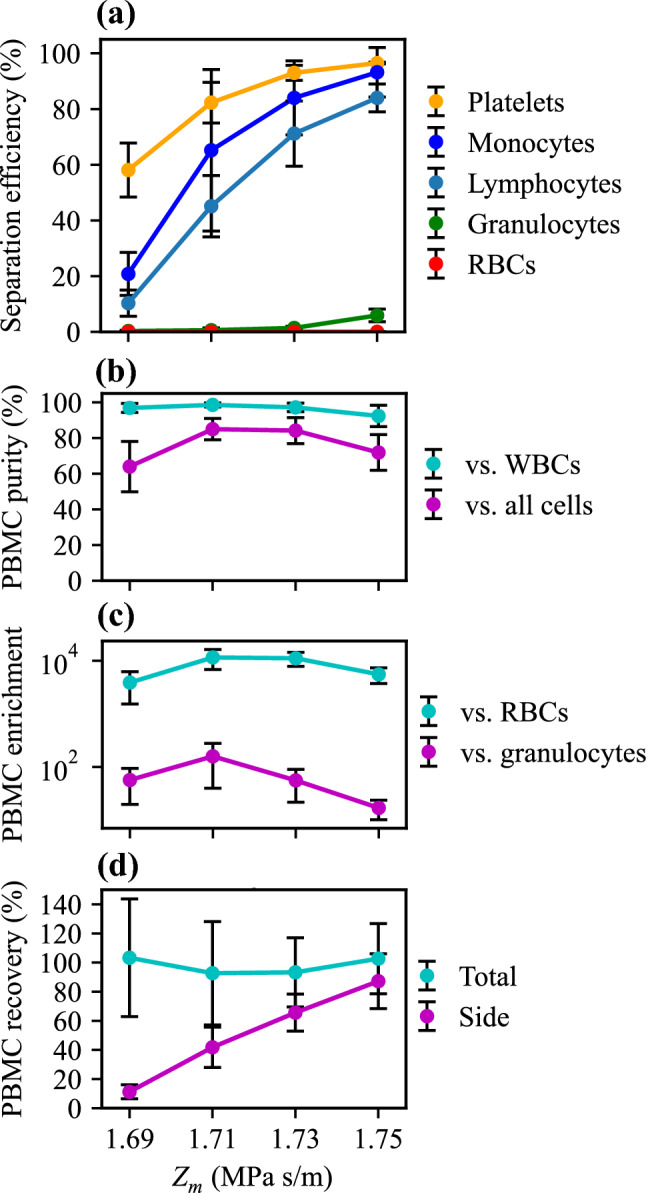
Barrier medium separation. (**a**) Separation efficiency, (**b**) output purity, (**c**) relative enrichment of PBMCs compared to other cells (log scale), and (**d**) PBMC recovery. Data points are average values of *n* = 5 experiments with ± one standard deviation.

The resulting separation efficiencies for platelets, granulocytes, monocytes, lymphocytes, and RBCs were measured for each cell type, Fig. 3a and Supplementary Figure S1. A higher PBMC separation efficiency is observed when increasing the acoustic impedance of the barrier medium. This agrees with the expectation that PBMCs are prevented from focusing into the central zone of the channel as the acoustic impedance of the central medium increases. The separation efficiencies for monocytes and lymphocytes suggest that monocytes require a slightly lower acoustic impedance barrier to remain in the side fraction compared to lymphocytes. This indicates that monocytes have lower acoustic impedance than lymphocytes, which is supported by previous measurements where these cell populations showed overlapping but slightly shifted acoustic impedance distributions^50^. In that study, PBMCs were measured to have effective acoustic impedances in the range of 1.65 to 1.71 MPa s/m, allowing a large fraction of these cells to penetrate barrier media in the two lowest measurement points, *i.e., Z*_*m*_ = 1.69 and 1.71 MPa s/m. The difference cannot be an effect of cell size since monocytes, which are in general larger than lymphocytes, would have migrated faster than lymphocytes into the central zone if they would have had identical acoustic properties and were not blocked by the barrier.

Even though a barrier medium of *Z*_*m*_ = 1.73 MPa s/m should have sufficiently high acoustic impedance to block PBMCs, it is not until *Z*_*m*_ = 1.75 MPa s/m that they are efficiently hindered. This observation can be attributed to the diffusion of the iodixanol molecules, which causes a gradual transition from low to high acoustic impedance when approaching the barrier. This effect makes it possible for some of the cells to penetrate inside the region of the flow that is later routed through the central outlet. A similar effect is observed for granulocytes, which start appearing in the side outlet due to being blocked, for *Z*_*m*_ = 1.75 MPa s/m. According to literature^50^, they should be nearly completely blocked in this medium, but due to the barrier degradation, the majority of granulocytes can still reach the central region of the flow.

To achieve high purity in the side fraction, a compromise must be made between blocking PBMCs while still enabling efficient transfer of RBCs and granulocytes through the barrier. Maximum PBMC purity was achieved for *Z*_*m*_ = 1.71 MPa s/m, Fig. 3b, but *Z*_*m*_ = 1.73 MPa s/m had only slightly lower purity and recovered more PBMCs, Fig. 3a. Since the barrier is not discrete, the optimal medium for separation will depend on the relative side and central flows, the total flow in the channel, and the amplitude of the acoustic field. A system of very high acoustic amplitude could be favorable in this regard since it could be operated at faster flow, and hence, the barrier will be more distinct, and overall, the separation would be less sensitive to variations.

The plot of PBMC purity relative to all the cells (excluding platelets) and relative to only the WBCs shows that RBCs are the main contaminant in the final product, Fig. 3b. However, the system is highly efficient in discarding RBCs for all tested barrier media, and the PBMC purity goes from initially approximately 1 PBMC in 2000 cells in whole blood to a final purity of more than 8 PBMCs out of every 10 cells in the output, comprising a 3.6 × 10^3^ to 1.1 × 10^4^-fold relative enrichment, Fig. 3c. This level of RBC depletion is high compared to most other microfluidic methods^34,42^, and in the range of what has been shown for Ficoll density centrifugation^42^.

The PBMC recovery shows that overall, very few cells are lost in the system in this approach, Fig. 3d. Values exceeding 100% is possible due to a large measurement uncertainty when measuring rare cells in diluted blood and since the input level is measured based on analyzing a single reference sample for each analyzed donor and day. Looking in the target outlet (side), PBMCs increase with increasing barrier medium acoustic impedance approaching 100% recovery for the highest impedance barrier.

**Figure 4:**
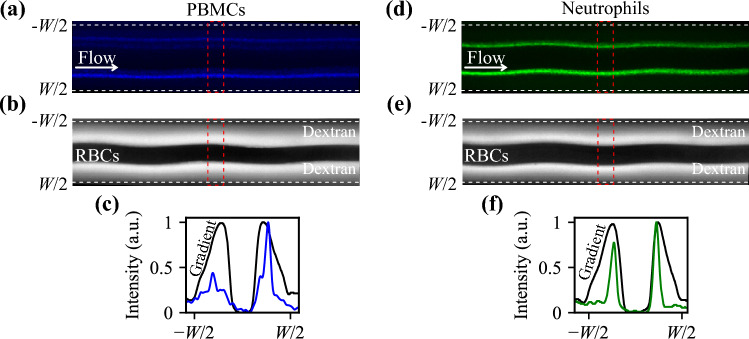
Cell arrangement at the end of the channel with a barrier medium of *Z*_*m*_ = 1.73 MPa s/m. (**a**) Overlay of 100 images containing PBMCs, and (**b**) the corresponding image of packed RBCs and barrier medium mixed with fluorescent dextran. (**c**) Resulting locations of PBMCs (blue) and barrier fluorescent dextran intensity (black). The presence of RBCs blocks the light in the central region. (**d**) Overlay of 100 images of neutrophils, (**e**) the corresponding image of packed RBCs and barrier medium mixed with fluorescent dextran. (**f**) Resulting locations of neutrophils (green) and barrier fluorescent dextran intensity (black). Intensities in (**c**) and (**f**) were analyzed in the region indicated by a red dashed box in (**a**), (**b**), (**d**), and (**e**).

To further investigate the cells’ arrangements in relation to RBCs at the end of the channel, for a barrier medium of *Z*_*m*_ = 1.73 MPa s/m, we imaged stained PBMCs, neutrophils (the major sub-group of granulocytes), and the barrier medium containing fluorescent dextran. At the inlet of the channel [Supplementery Figure S2], the barrier medium occupies the channel center, the blood stays on both sides, and all components (fluids and cells) settle according to their effective acoustic impedance during the passage. Figure 4a–c confirm that at the end of the channel, PBMCs, with a lower acoustic impedance than RBCs, are separated from the packed RBCs in the center of the channel with only a small fraction reaching the interface. Neutrophils, on the other hand, are able to penetrate the RBC interface to some degree, Fig. 4d–f, and [Supplementery Figure S3] shows that the location of cells converges when the applied acoustic field amplitude increases.

### Pre-mix PBMC enrichment

**Figure 5:**
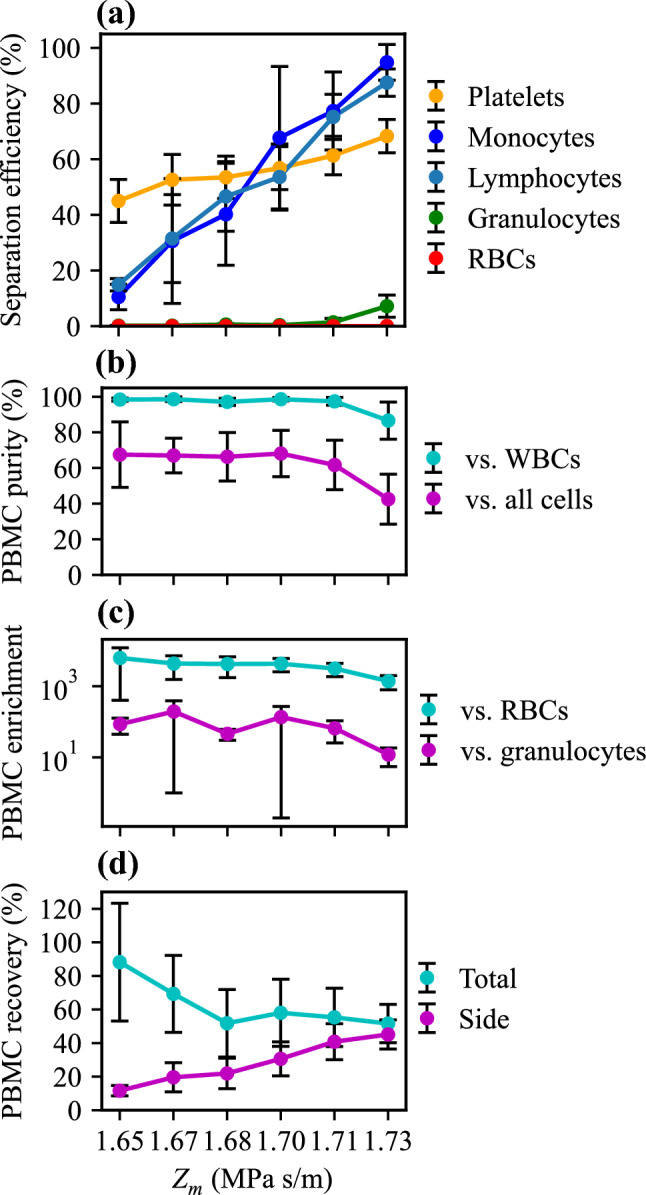
Pre-mix approach separation. (**a**) Separation efficiency, (**b**) output purity, (**c**) relative enrichment of PBMCs compared to other cells (log scale), and (**d**) PBMC recovery data points are average values of *n* = 5 experiments with ± one standard deviation.

Using the pre-mix approach, we investigated the effect of increasing the acoustic impedance of the blood plasma by mixing the blood with iodixanol. In this approach, cells are distributed across the entire channel cross-section at the inlet, which means that PBMCs must have sufficient negative acoustic contrast factor to migrate all the way to the side outlet before exiting the chip. Therefore, the range of working solutions was extended to include 36% and 39% iodixanol. Figure 5a and Supplementary Figure S4 show that the pre-mix approach achieves monocyte and lymphocyte separation efficiencies higher than 80%, which is comparable to standards within blood fractionation today. The highest PBMC purity relative to all cells (excluding platelets) was overall very high and only drops for *Z*_*m*_ = 1.735 MPa s/m due to the drop in acoustic contrast for RBCs and granulocytes preventing them from being completely focused to the central region, Fig. 5b. The relative enrichment compared to RBCs ranges from 1.3 × 10^3^ to 8.9 × 10^3^-fold, Fig. 5c.

The PBMC recovery shows that more cells are lost in the system in the pre-mix approach compared to the barrier approach, and interestingly the recovery drops with increasing acoustic impedance, Fig. 5d. This can be caused by PBMCs’ acoustic contrast being increasingly negative for higher acoustic impedance. Negative contrast cells are pushed to the walls of the channel where the flow velocity is low and they may get arrested in the chip. Alternatively, it can have to do with an increased buoyancy of cells for increasing acoustic impedance. Since sample is drawn from the bottom of the test tube, cells of high buoyancy may never reach the outlet container since the last volume of sample to enter the system is never retrieved.

**Figure 6:**
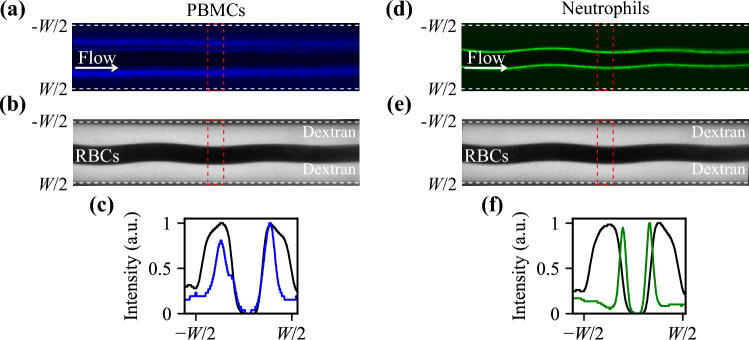
Cell arrangement at the end of the channel for a scenario where the blood was pre-mixed with the working medium to have *Z*_*m*_ = 1.703 MPa s/m. (**a**) Overlay of 100 images containing PBMCs, and (**b**) the corresponding image of packed RBCs and density-modified plasma mixed with fluorescent dextran. (**c**) Resulting location of PBMCs (blue) and fluorescent dextran intensity in the modified plasma (black). The presence of RBCs blocks the light in the central region. (**d**) Overlay of 100 images of neutrophils, (**e**) the corresponding image of packed RBCs surrounded by density-modified plasma with fluorescent dextran. (**f**) Resulting locations of neutrophils (green) and fluorescent dextran intensity in the modified plasma (black). Intensities in (**c**) and (**f**) were analyzed in the region indicated by a red box in (**a**), (**b**), (**d**), and (**e**).

Figure 6 shows the arrangement of PBMCs, RBCs, and neutrophils at the end of the channel for a blood sample pre-mixed with the working medium of *Z*_*m*_ = 1.703 MPa s/m. The fluorescence intensity plot in Fig. 6c indicates that PBMCs do not acquire enough negative contrast to be pushed all the way to the side walls. Neutrophils, however, penetrate the packed RBCs and can be collected in the central outlet. [Supplementery Figure S5] shows that the location of cells converges when the applied acoustic field amplitude increases.

For all analyzed samples, the number of dead cells was counted by flow cytometry after propidium iodide staining. Only 2% of the cells were found dead by this method, which agrees with previous reports demonstrating maintained proliferation, activation ability, and cell function after acoustic processing for several different cell types ^58,59^.

## Discussion

Both methods yield similar results regarding separation efficiency, but with the barrier approach, the maximum purity and relative enrichment of PBMCs are higher than for the pre-mix approach. To achieve high separation efficiency in the pre-mix approach, PBMCs must be pushed out from the central region by achieving negative contrast through increasing *Z*_*m*_. This leads to low acoustic contrast for RBCs and granulocytes, causing less efficient removal of these cells. In contrast, in the barrier approach, RBCs and granulocytes are initially migrating in plasma with comparably higher acoustic contrast and mobility, which enables them to reach and cross the barrier before exiting the channel.

The difference between individual lymphocyte and monocyte populations seems to be smaller in the pre-mix separation method, Fig. 5a**,** compared to the barrier approach, Fig. 3a. In the barrier medium approach, cells move in the gradient until they reach their iso-acoustic point, which is determined by their effective acoustic impedance. Since lymphocytes have slightly higher effective acoustic impedance, they move further and have a higher risk of ending up in the central outlet. In the pre-mix method, however, where no impedance gradient is present, the separation is governed by the acoustically induced velocity of the cells, which depends on both size and acoustic contrast, Eq. (3). The smaller size of lymphocytes and their higher acoustic impedance may thus lead to a similar velocity as for the larger but less compact monocytes.

In line with most separation methods, there is a trade-off between purity and separation efficiency. Here, we achieved 88% and 85% separation efficiencies for the pre-mix and barrier medium methods with 42% and 72% purity, respectively. However, purities up to 68% and 85% were achieved when the media acoustic impedances were optimized for purity rather than separation efficiency. This is comparable to most presented microfluidic techniques even though this method is processing cells at a much higher rate^42^.

Both presented methods process the blood with comparable throughput. The 60% dilution of the blood sample in the pre-mix approach leads to a larger processed sample volume. This is compensated by a higher sample flow rate which is possible since there is no central barrier medium, thus maintaining a similar retention time and throughput of cells in the separation channel for the two methods (see Table 2).

The variation in separation efficiency for repeated experiments can be caused by variations between donors, such as hematocrit and plasma composition. Flow and acoustic field instabilities are other sources that affect separation quality. Another source of instability can be observed in Figs. 4 and 6. The sound field is not perfectly invariant along the channel which can lead to an asymmetric distribution of cells at the outlet. The effect tends to decay near the outlet where the acoustic field drops off.

When density centrifugation is employed for PBMC isolation, platelets remain in the PBMC fraction to a large extent and are typically removed through an additional centrifugation step. Similarly, platelets also remain in the PBMC fraction after the acoustophoretic process presented here. The separation of platelets from PBMCs using acoustophoresis has previously been presented^45^, where > 90% removal was achieved and is thus not investigated further herein. The possibility of combining the two acoustic separation steps to obtain a platelet-free PBMC sample can be considered feasible.

Exposure of blood cells to gradient media containing agents that increase the density, such as iodixanol, polysaccharides, or polymer-coated colloidal silica particles has been reported to be associated with altered properties and function^60–63^. Therefore, prolonged exposure at high concentrations should be avoided. In that regard, the barrier approach can be considered to be the better choice of the two evaluated methods, since the PBMCs are untouched until entry in the device. In addition, the average iodixanol concentration in the PBMC outlet is lower in the barrier approach than in the pre-mix approach since that outlet contains mostly blood plasma.

This study was limited to varying the acoustic properties of the suspending medium while keeping important factors such as the acoustic amplitude and flow rate fixed. The flow rate, and thus the retention time, and the acoustic field will affect if cells have sufficient time to reach their equilibrium positions during separation. Further, the barrier concentration gradient will be affected by the retention time which will influence the performance. Therefore, further parametric studies should be undertaken to establish if the current results represent optimal separation in the two approaches.

## Conclusion

We propose two approaches, barrier and pre-mix, to purify PBMCs from whole blood using acoustic focusing and dense media containing iodixanol. When comparing the two methods, the barrier medium principle, where PBMCs are blocked by a high acoustic impedance medium, shows slightly better performance in terms of the achieved sample purity. The advantage of the pre-mix method lies in its simplicity, requiring only one inlet while still achieving high separation efficiency. Compared to previously proposed microfluidics methods to enrich PBMCs from blood, only inertial focusing^19^ achieves comparable throughput in terms of whole blood equivalent volume flow per processing channel, while acoustophoresis using density medium yielded better separation performance and enabled direct input of whole blood^42^.

### Supplementary Information

Supplementary Information.

## Data Availability

Data and computer code generated during the current study is available from the corresponding authors upon reasonable request.
