# Supplementary material for: Label-free separation of peripheral blood mononuclear cells from whole blood by gradient acoustic focusing
Source: Sci Rep. 2024 Apr 16;14:8748. doi: 10.1038/s41598-024-59156-7 (PMC11021555; doi:10.1038/s41598-024-59156-7)
Supplement: Supplementary file 1 — Supplementary Information. [file 41598_2024_59156_MOESM1_ESM.pdf]

Supporting information for paper:

Label-Free Separation of Peripheral Blood Mononuclear Cells from Whole Blood by Gradient Acoustic Focusing

Julia Alsved[a†], Mahdi Rezayati Charan[b†], Pelle Ohlsson[a,b], Anke Urbansky[a], Per Augustsson[b]*

[a] AcouSort AB, Medicon Village, S-223 81 Lund, Sweden

[b] Department of Biomedical Engineering, Lund University, Ole Römers väg 3, 22363 Lund, Sweden

* per.augustsson@bme.lth.se

[†] Equal contribution

S1. RBCs in the side fraction

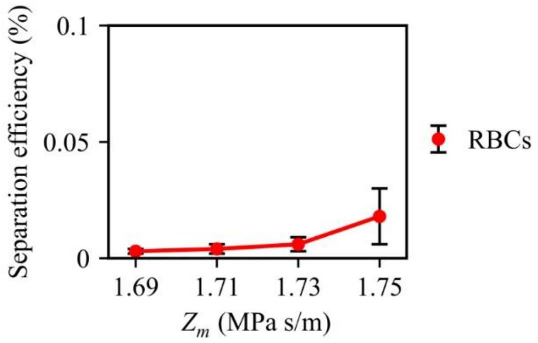

Figure S1. Zoom in of the separation efficiency of RBCs in the barrier medium method.

S2. Configuration of RBCs and barrier medium at the inlet

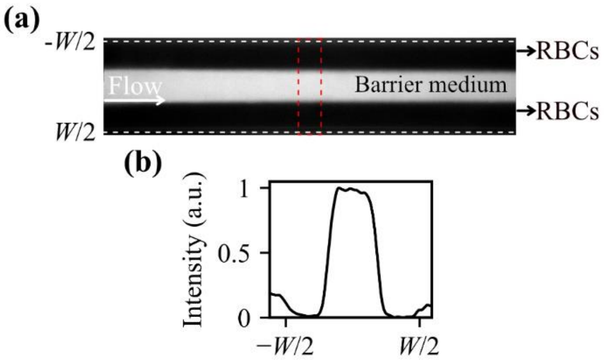

Figure S2. (a) An image of the channel's inlet where the barrier medium containing dextran fluorescent tracer occupies the center, and whole blood is laminated on both sides, and (b) the corresponding intensity analysis of the channel's cross-section at the center marked with a red dashed rectangle.

S3. Cells positioning at different applied voltages in the barrier medium setting

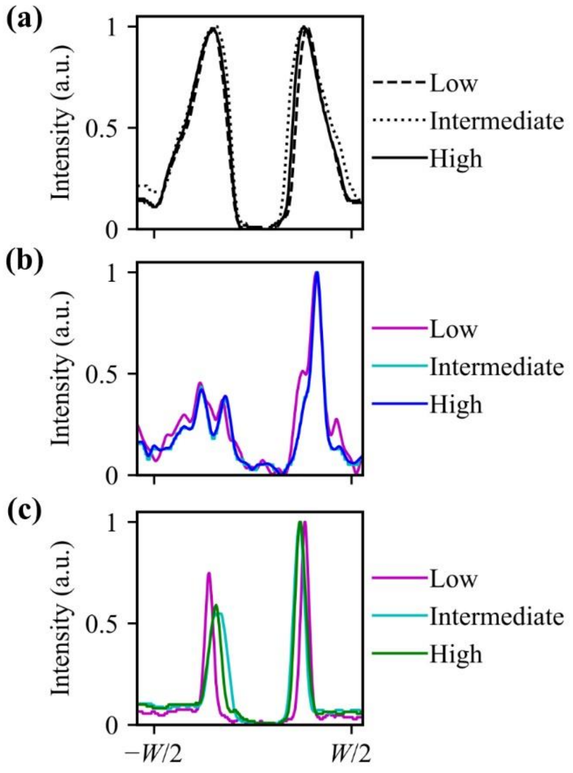

Figure S3. Intensity analysis of (a) packed RBCs and barrier medium, (b) PBMCs, and (c) neutrophils at the end of the channel for three different applied voltages. The analysis was done for a cross-section at the center of the imaging field.

S4. RBCs in the side fraction

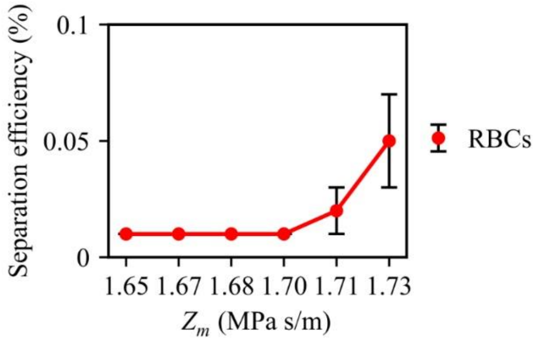

Figure S4. Zoom in of the separation efficiency of RBCs in the pre-mix method.

S5. Cells positioning at different applied voltages in the pre-mix setting

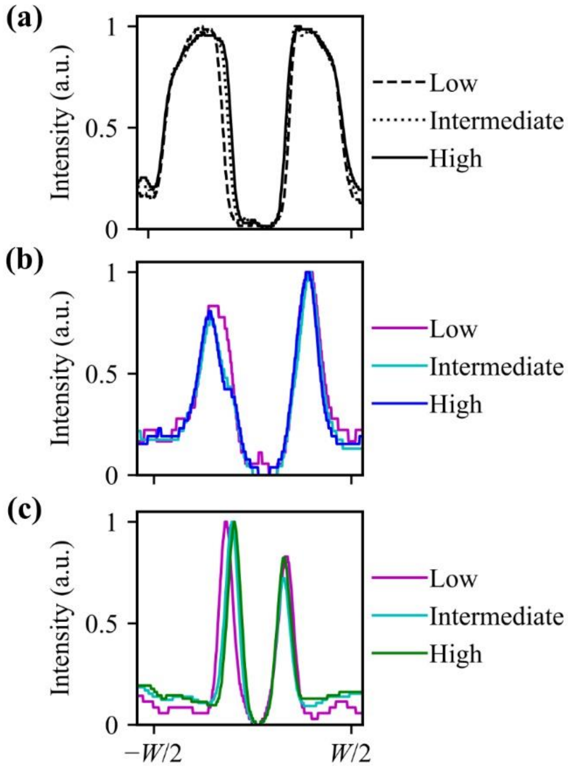

Figure S5. Intensity analysis of (a) packed RBCs and density-modified medium, (b) PBMCs, and (c) neutrophils at the end of the channel for three different applied voltages. The analysis was done for a cross-section at the center of the imaging field.
